# Combining features selection strategy and features fusion strategy for SPAD estimation of winter wheat based on UAV multispectral imagery

**DOI:** 10.3389/fpls.2024.1404238

**Published:** 2024-05-10

**Authors:** Xiangxiang Su, Ying Nian, Hiba Shaghaleh, Amar Hamad, Hu Yue, Yongji Zhu, Jun Li, Weiqiang Wang, Hong Wang, Qiang Ma, Jikai Liu, Xinwei Li, Yousef Alhaj Hamoud

**Affiliations:** ^1^ College of Resource and Environment, Anhui Science and Technology University, Fengyang, China; ^2^ College of Environmental, Hohai University, Nanjing, China; ^3^ Anhui Engineering Research Center of Smart Crop Planting and Processing Technology, Fengyang, China; ^4^ Anhui Province Agricultural Waste Fertilizer Utilization and Cultivated Land Quality Improvement Engineering Research Center, Anhui Science and Technology University, Fengyang, China; ^5^ College of Hydrology and Water Resources, Hohai University, Nanjing, China

**Keywords:** UAV, winter wheat, SPAD, features selection strategy, features fusion strategy

## Abstract

The Soil Plant Analysis Development (SPAD) is a vital index for evaluating crop nutritional status and serves as an essential parameter characterizing the reproductive growth status of winter wheat. Non-destructive and accurate monitorin3g of winter wheat SPAD plays a crucial role in guiding precise management of crop nutrition. In recent years, the spectral saturation problem occurring in the later stage of crop growth has become a major factor restricting the accuracy of SPAD estimation. Therefore, the purpose of this study is to use features selection strategy to optimize sensitive remote sensing information, combined with features fusion strategy to integrate multiple characteristic features, in order to improve the accuracy of estimating wheat SPAD. This study conducted field experiments of winter wheat with different varieties and nitrogen treatments, utilized UAV multispectral sensors to obtain canopy images of winter wheat during the heading, flowering, and late filling stages, extracted spectral features and texture features from multispectral images, and employed features selection strategy (Boruta and Recursive Feature Elimination) to prioritize sensitive remote sensing features. The features fusion strategy and the Support Vector Machine Regression algorithm are applied to construct the SPAD estimation model for winter wheat. The results showed that the spectral features of NIR band combined with other bands can fully capture the spectral differences of winter wheat SPAD during the reproductive growth stage, and texture features of the red and NIR band are more sensitive to SPAD. During the heading, flowering, and late filling stages, the stability and estimation accuracy of the SPAD model constructed using both features selection strategy and features fusion strategy are superior to models using only a single feature strategy or no strategy. The enhancement of model accuracy by this method becomes more significant, with the greatest improvement observed during the late filling stage, with R^2^ increasing by 0.092-0.202, root mean squared error (RMSE) decreasing by 0.076-4.916, and ratio of performance to deviation (RPD) increasing by 0.237-0.960. In conclusion, this method has excellent application potential in estimating SPAD during the later stages of crop growth, providing theoretical basis and technical support for precision nutrient management of field crops.

## 1 Introduction

As a principal food crop worldwide, wheat provides vital carbohydrate sustenance to millions of people, holding an essential position in addressing global food security (Asseng et al., 2011). The Soil Plant Analysis Development (SPAD) indicator, which represents the relative chlorophyll content in leaves (Netto et al., 2005), is intimately connected with crop nutritional status, serving as a significant index of crop growth and development (Gitelson et al., 2005; Zhang et al., 2019; Liu et al., 2020). Consequently, the accurate and efficient acquisition of SPAD information for winter wheat is of paramount importance for field management decisions and crop nutrition monitoring. The conventional approaches for obtaining crop chlorophyll content primarily rely on destructive field sampling and laborious chemical analysis in the laboratory. These approaches are not only time-consuming and labor-intensive but also incur significant costs (Sampson et al., 2003). Existing research has demonstrated a close correlation between data obtained from the handheld SPAD-502 chlorophyll meter and the chlorophyll content analyzed chemically in a laboratory setting (Rigon et al., 2016). Although the SPAD acquisition method offers more convenience than traditional chlorophyll measurement techniques and allows for non-destructive sampling (Uddling et al., 2007), its limitations in measurement points entail certain labor costs, resulting in small operational scales, low efficiency, and difficulty in meeting the demands for large-scale acquisition of crop chlorophyll content information (Wang J et al., 2021; Wu et al., 2023). Therefore, there is a pressing need for a method that can rapidly, non-destructively, and accurately obtain crop SPAD information.

High-throughput phenotyping (HTP) technology has made this work possible, especially the unmanned aerial vehicle (UAV) phenotyping platform equipped with high-performance image sensors (Guo et al., 2021). Its advantages of high spatio-temporal resolution, flexibility, and easy operation have been widely applied to crop growth monitoring (Liu J et al., 2022; Yin et al., 2022; Fan et al., 2023). With the development of sensor technology, RGB sensors (Vélez et al., 2022), multispectral sensors (Impollonia et al., 2022), hyperspectral sensors (Zhou X et al., 2023), and lidar (Jimenez-Berni et al., 2018) have provided substantial data support for crop phenotyping, intelligent breeding, and more (Jin et al., 2015). Although RGB sensors are cost-effective, the spectral information they can acquire is limited, and the limited number of spectral bands often struggle to accurately capture the complete physiological and biochemical information of crops (Feng et al., 2022). Hyperspectral sensors and lidar offer high data accuracy, but due to their high cost, they are not suitable for widespread application in field crop monitoring (Madec et al., 2017; Fan et al., 2022). Therefore, the use of a lightweight, cost-effective UAV equipped with a multispectral sensor to capture remote sensing information of the winter wheat canopy for SPAD estimation becomes particularly essential.

Currently, spectral features of UAV imagery (vegetation indices, bands reflectance) have been extensively utilized in the field of precision agriculture for estimating crop physiological parameters (Luo et al., 2022), such as Leaf Area Index (LAI) (Qiao et al., 2022; Zhou et al., 2022), Above-Ground Biomass (AGB) (Han et al., 2022; Yue et al., 2023), SPAD (Wang J et al., 2021; Yang et al., 2021), and nitrogen content (Yang et al., 2019; Xu et al., 2023). However, under high canopy cover in the later stages of crop growth, vegetation indices can be affected by spectral saturation effects (Mutanga and Skidmore, 2004; Yue et al., 2019; Mutanga et al., 2023). Moreover, during the reproductive growth stage of crops, the emergence of crop spikes further increases the spectral mixing effect, reducing the sensitivity of vegetation indices (Jay et al., 2019; Wang W et al., 2021). It is challenging to establish a reliable SPAD estimation model using only spectral features during the reproductive growth stage. Texture features, as remote sensing information reflecting image grayscale attributes, color, and spatial structure, can not only characterize differences in canopy image spatial structure arrangement caused by crop growth and development (Liu et al., 2022a) but also reflect changes in crop leaf color due to different treatments such as varieties, fertilizers, and density (Li R. et al., 2022). Texture features have the potential to be combined with spectral features (Fu et al., 2021), and features fusion can reduce the impact of spectral saturation effects to enhance the accuracy of estimation models (Li et al., 2020). However, with the increase of features, while improving model accuracy, there may be information redundancy (Zheng et al., 2020), increasing data complexity. Therefore, to eliminate this redundancy, selecting appropriate features is extremely necessary for optimizing model structure and accuracy.

Features selection plays a pivotal role in machine learning, as it directly determines the input features of the prediction model, influencing its performance and effectiveness. RFE method is widely used in features selection nowadays (Zhu et al., 2020; Shao et al., 2022; Yang et al., 2022). This method is based on multiple rounds of training using a specific algorithm model. It constructs new feature subsets by removing particular variables and conducts the next round of training based on the new feature set (Chen et al., 2021; Rasel et al., 2021). This process is repeated until the model performs optimally. In recent years, the Boruta features selection method has quickly gained popularity due to its low operational cost and speed (Shu et al., 2021; Tatsumi et al., 2021; Shu et al., 2023). The goal of Boruta is to filter out all feature sets related to the target parameter. It allows a more comprehensive understanding of the factors influencing the target parameter, thereby effectively performing features selection (Kursa and Rudnicki, 2010).

Considering the impact of spectral saturation in the later stages of crop growth, features selection and features fusion have the potential to enhance the performance of prediction models. Identifying the remote sensing features that influence the SPAD of winter wheat in the later stages of growth and jointly constructing a high-accuracy SPAD model will be a key focus of future research. Therefore, this study utilizes a UAV equipped with a multispectral sensor to capture high-resolution images of the winter wheat canopy, and employs image processing technology to extract the spectral and texture features of the wheat, and utilizes two features selection methods (Boruta and RFE) to explore the potential of spectral and texture features in predicting SPAD during the later stages of winter wheat growth. We also develop a winter wheat SPAD monitoring model that combines features selection and features fusion strategy. The objectives of this study are: (1) to identify the spectral and texture features sensitive to SPAD during the reproductive growth stage of winter wheat; (2) to evaluate the performance of the winter wheat SPAD prediction model under features selection strategy; and (3) to explore the potential of combining features selection and features fusion strategy to estimate SPAD in the later stages of winter wheat growth.

## 2 Materials and methods

### 2.1 Field experimental design

**Figure 1:**
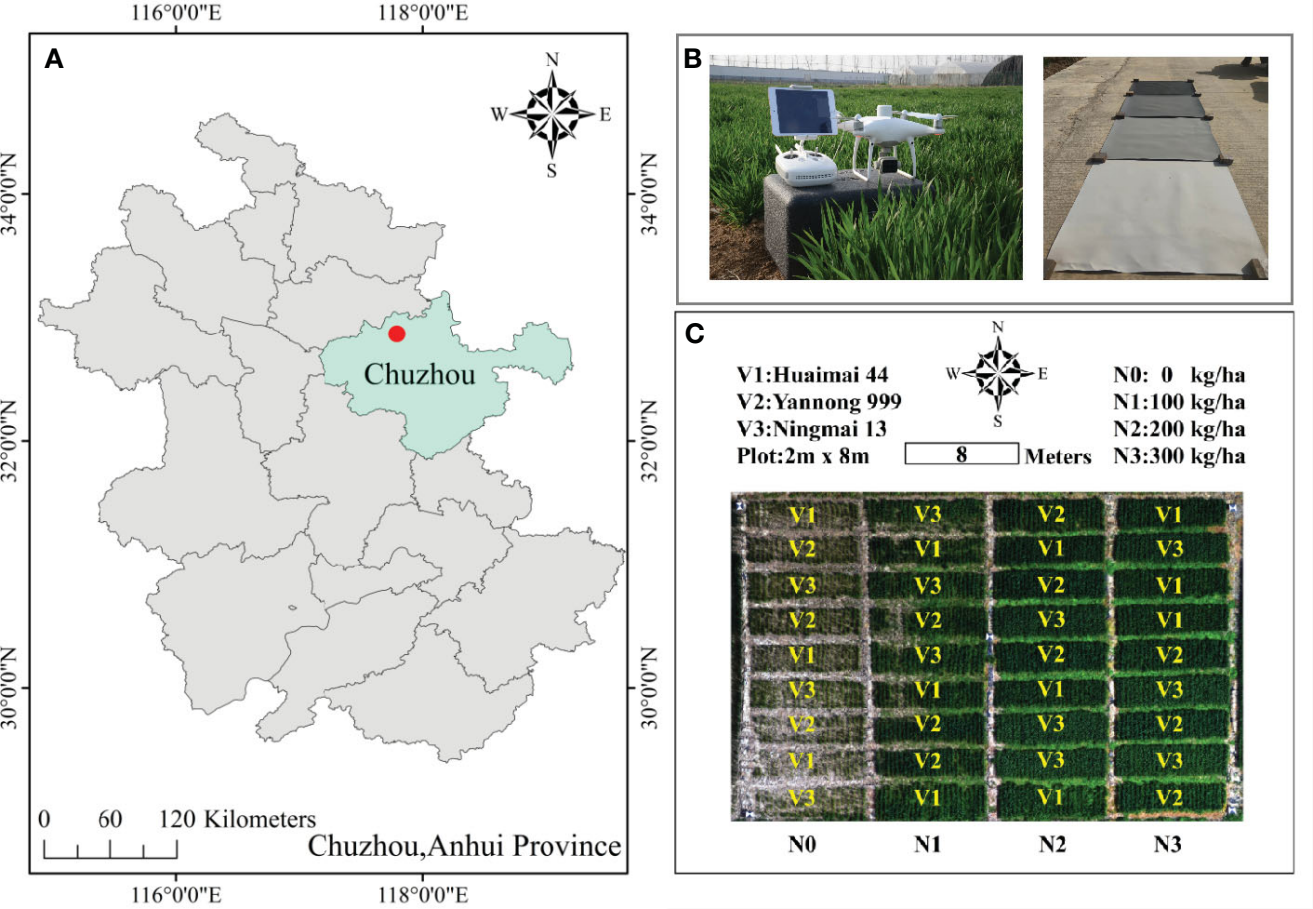
Study area **(A)**. The experiment field was in Chuzhou, Anhui province, China. **(B)**, the unmanned aerial vehicle (UAV) for acquiring low-altitude remote sensing data and plates that correct 5%, 10%, 20% and 40% reflectivity of calibrate differences and **(C)** field experimental design.

The winter wheat experiment took place in Chuzhou City, located in Anhui Province, China (32°48′52″N,117°46′7″E) from 2020 to 2021 (**Figure 1A**). This region, positioned in the mid-lower reaches of the Yangtze River, experiences a subtropical monsoon climate. It is characterized by a moist weather pattern, distinct four seasons, an average annual temperature of 15.4°C, and an annual rainfall between 1000-1100mm. The region records an average of 144 rainy days per year and approximately 210 frost-free days annually.

The field experiment consisted of 36 plots, each measuring 16m² (2×8 m). It involved three winter wheat varieties with stable high-yield potential (V1: Huaimai 44, V2: Yannong 999, and V3: Ningmai 13) and four levels of nitrogen fertilizer application (N0-N3: 0, 100, 200, 300 kg/ha), with each treatment replicated three times (**Figure 1C**). The fertilization scheme involved the application of phosphorus (P=90kg/ha) and potassium (K=135 kg/ha) as base fertilizers before sowing, with a 6:4 ratio of nitrogen fertilizer applied pre-sowing and at the jointing stage. The sowing date was November 7, 2020, with the winter wheat manually strip-sown at a row spacing of 30 cm. The harvest took place on June 3, 2021, and other field management measures were based on local high-yield cultivation practices. Throughout the entire growth stage of the winter wheat, there were no occurrences of diseases, pests, or weed hazards, and the field environment was good, with no instances of drought or waterlogging.

### 2.2 UAV-based data collection and pre-processing

The experiment utilized a Phantom 4 Multispectral (P4M) RTK (DJI Technology Co., Shenzhen, China) UAV to collect multispectral (MS) data during the wheat heading stage on April 18, 2021, the flowering stage on April 29, 2021, and the late filling stage on May 24, 2021. The UAV is equipped with five monochrome sensors, each with 2.08 million pixels: Blue, Green, Red, Red edge, and NIR, with center wavelengths of 450 ± 16nm, 560 ± 16nm, 650 ± 16nm, 730 ± 16nm, and 840 ± 26nm, and bandwidths of 20nm, 20nm, 10nm, 10nm, 40nm, respectively. It is also equipped with a real-time kinematic (RTK) system, which allows for vertical positioning accuracy of ±1.5 cm and horizontal positioning accuracy of ±1cm, enabling the P4M to obtain high-precision spectral and texture information (Oktay and Coban, 2017). Flights were conducted between 11:00 and 13:00 on clear, windless days with stable solar radiation intensity. Four radiometric calibration panels with known reflectance were deployed on the ground for radiometric calibration during subsequent data processing (**Figure 1B**). The DJI GS PRO software (https://www.dji.com/cn/ground-station-pro/) was used to pre-plan the flight route, relying on the UAV’s autopilot system to execute the predefined flight plan (Şahin et al., 2022). Each flight lasted 20 minutes, with a flight altitude of 30 m, a flight speed of 2m/s, forward and side overlap of 90% and 85% respectively, and an image resolution of 1600×1300 pixels.

The UAV MS images collected at each growth stage were imported into the PIX4Dmapper software (version 4.4.12, Pix4D SA, Prilly, Switzerland) for image stitching. The initial step involved aligning the images using the feature point matching algorithm. Subsequently, a dense point cloud and texture mesh were generated by utilizing both the UAV image data and location information. To improve data quality, this study carried out radiometric correction on the MS images using the empirical line method (ELM) and the image information of ground radiometric calibration panels with known reflectance (Di Gennaro et al., 2022). A shapefile was generated using ArcGIS 10.2 (Environmental Systems Research Institute, Inc, RedLands, CA, USA), and plot areas were delineated based on the orthoimage of the experimental area.

### 2.3 SPAD data collection

Field measurements at each stage were conducted prior to the UAV flights on the same day, with chlorophyll values based on SPAD determined for winter wheat across the 36 plots. Winter wheat SPAD was measured using a SPAD-502 chlorophyll meter (Konica Minolta Optics Inc., Osaka, Japan) (Jiang et al., 2022). For each plot, three average-growth wheat plants were selected, and SPAD readings were taken at the 1/6, 3/6, and 5/6 length of the flag leaves. The average of the 9 SPAD values in the sample plot was used as the actual SPAD measurement in the sample plot. A total of 324 times were collected in each growth stage, and the measured SPAD data of 36 sample plots were calculated.

### 2.4 Extraction of features from UAV imagery

#### 2.4.1 Spectral features extraction

This study used band reflectance and vegetation indices as spectral features (SF). Vegetation indices are obtained by calculations or combinations of characteristic bands, providing robust vegetation information factors and enhancing, to a certain extent, the expressive ability of remote sensing data (Bai et al., 2022). The reflectance of the five original bands from the wheat canopy during the heading, flowering, and late filling stages was obtained from the MS images for vegetation index calculation. This study selected a total of 30 widely-used spectral features for monitoring crop growth and evaluating parameters These features include five original bands (I), seven with only the visible bands (II), five with the RE band but without NIR band (III), eight with the NIR band but without RE band (IV), and five with both the NIR and RE bands (V) (**Table 1**).

**Table 1:** Types and formulas of spectral features extracted from MS images based on UAV.

Component	Variable	Formulation	References
The original bands	Blue	B	–
	Green	G	–
	Red	R	–
	Red edge	RE	–
	Near-infrared	NIR	–
With only the Visible bands	CI	(R-B)/R	(Kumar et al., 2015)
	NGBDI	(G-B)/(G+B)	(Hunt et al., 2005)
	RBI	R/B	(Peña Barragán et al., 2007)
	NPCI	(R-B)/(R+B)	(Peñuelas et al., 1994)
	WI	(G-B)/(R-G)	(Woebbecke et al., 1995)
	ExB	1.4*B-G	(Mao et al., 2003)
	GBI	G/B	(Sellaro et al., 2010)
With the Red edge band but without NIR band	PSRI	(R-G)/RE	(Ren et al., 2017)
	LIC3	B/RE	(Lichtenthaler et al., 1996)
	DCabCxc	R/G*3*RE	(Poblete et al., 2020)
	GM1	RE/G	(Gitelson and Merzlyak, 1996)
	NDREI	(RE-G)/(RE+G)	(Hassan et al., 2018)
With the NIR band but without Red edge band	DVI	NIR-R	(Richardson and Everitt, 1992)
	mSR	(NIR-B)/(RE-B)	(Datt, 1999)
	CVI	NIR*R/(G*G)	(Datt et al., 2003)
	SIPI	(NIR-B)/(NIR-R)	(Hunt et al., 2011)
	GCVI	NIR/G-1	(Gitelson et al., 2003)
	ARI3	NIR*(1/G-1/R)	(Gitelson et al., 2001)
	BDVI	NIR-B	(de Castro et al., 2012)
	NDVI	(NIR-R)/(NIR+R)	(Bannari et al., 1995)
With the NIR band and Red edge band	NDRE	(NIR-RE)/(NIR+RE)	(Le Maire et al., 2008)
	LCI	(NIR-RE)/(NIR+R)	(Hunt et al., 2011)
	MTCI	(NIR-RE)/(RE-R)	(Jurgens, 1997)
	DATT	(NIR-RE)/(NIR-R)	(Datt, 1999)
	mNDblue	(B-RE)/(NIR+B)	(Jay et al., 2017)

#### 2.4.2 Texture features extraction

As a common means of characterizing image information, texture reflects important information such as surface structure and spatial arrangement in the image without relying on brightness. It generally uses high-energy narrow peaks in the spectrum to detect the periodicity of the image (Bharati et al., 2004; Pu and Cheng, 2015). This study uses the Gray Level Co-occurrence Matrices (GLCM) method to extract the texture information of B, G, R, RE and NIR bands in winter wheat canopy MS images. GLCM requires user-defined parameters such as window size and orientation. The parameters for extracting GLCM textures in this study are as follows: a window size of 3 pixels by 3 pixels, an extraction direction set at 45°, and grayscale quantization levels defaulted to 64. The texture information includes Mean (Me), Variance (Va), Homogeneity (Ho), Contrast (Cn), Dissimilarity (Di), Entropy (En), Second moment (Se) and Correlation (Cr), a total of eight indicators (**Table 2**). In order to obtain more texture information for feature selection, the maximum value (MAX), minimum value (MIN), mean value (MEAN) and standard deviation (SD) of the GLCM index in each sample area were calculated, resulting in a total of 5(bands) × 8(GLCM indices) × 4(statistical metrics) = 160 texture metrics.

**Table 2:** Formulas of texture features extracted from MS images based on GLCM.

Texture features	Name	Formula	Description
Me	Mean	$Me=\sum_{i=0}^{N-1}\sum_{j=0}^{N-1}i*\text{p}(i,j)$	The mean value in the texture
Va	Variance	$V\text{a}=\sum_{i}\sum_{j}{\left(i-u\right)}^{2}\text{p}(i,j)$	the size of the texture change
Ho	Homogeneity	$Ho=\sum_{i}\sum_{j}\frac{1}{1+{\left(i-j\right)}^{2}}\text{p}(i,j)$	The homogeneity of grey level in the texture
Cn	Contrast	$C\text{n}=\sum_{\text{n}=0}^{{\text{N}}_{\text{g}}-1}{\text{n}}^{2}\left\{\begin{array}{c} \sum_{\text{i}=1}^{{\text{N}}_{\text{g}}}\sum_{\text{j}=1}^{{\text{N}}_{\text{g}}}\text{p}(i,j)\\ |i-j|=n \end{array}\right\}$	The clarity in the texture
Di	Dissimilarity	$Di=\sum_{\text{n}=1}^{{\text{N}}_{\text{g}}-1}\text{n}\left\{\begin{array}{c} \sum_{\text{i}=1}^{{\text{N}}_{\text{g}}}\sum_{\text{j}=1}^{{\text{N}}_{\text{g}}}\text{p}(i,j)\\ |i-j|=n \end{array}\right\}$	Same as contrast, The similarity of the pixels in the texture
En	Entropy	$En=-\sum_{i}\sum_{j}\text{p}(i,j)\log(\text{p}(i,j))$	The diversity of the pixels in the texture
Se	Second moment	$Se=\sum_{i}\sum_{j}\{p(i,j){\}}^{2}$	The uniformity of greyscale in the texture
Cr	Correlation	$C\text{r}=\frac{\sum_{i}\sum_{j}(i,j)\text{p}(i,j)-\mu_{i}\mu_{j}}{\sigma_{i}\sigma_{j}}$	The consistency in the texture

The parameters *u_i_*, *u_j_*, *σ_i_*, and *σ_j_* represent the average and standard deviation of the row and column sums of the GLCM.

### 2.5 Features selection strategy and features fusion strategy

**Figure 2:**
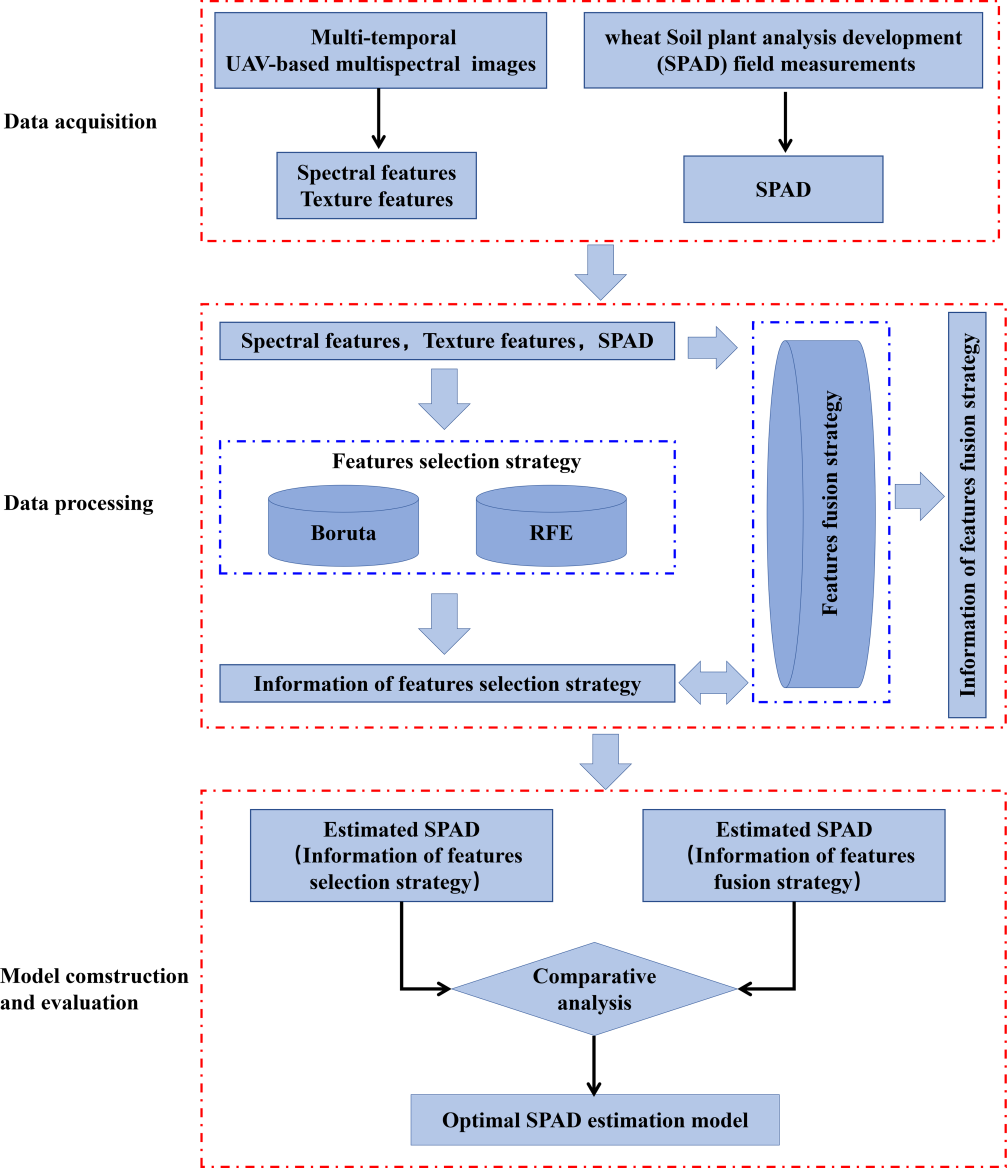
Flowchart of features selection strategy and features fusion strategy applied in this experimental study.

UAV multispectral remote sensing data was analyzed in order to identify the most effective approach to maximize improvements in SPAD estimates during later stages of growth (**Figure 2**). The features selection strategy is used to select SF and TF that are sensitive to SPAD. SPAD prediction model is further constructed to check the difference between implementing and not implementing the features selection strategy, different types of feature subsets are fused in the features fusion strategy to construct SPAD estimation model together.

#### 2.5.1 Features selection

In this study, two features selection methods were selected as features selection strategy: Recursive feature elimination (RFE) and Boruta. The RFE algorithm is a greedy search algorithm that incrementally selects the optimal features based on the model’s performance. Initially, RFE requires the selection of a specific machine learning model to serve as the estimator, such as linear regression, support vector machines, and random forests (RF). The RFE algorithm first uses all features to train the model, calculates the importance of each feature and sorts it, and uses each feature subset to train the model, compares the model results obtained from each subset, and then recalculates the model based on specific features. Train the model and repeat this process until the optimal feature combination is selected to maximize model performance (Kuhn, 2008). Ultimately, RFE yields a subset of features that the algorithm deems the most representative and significant. By employing this recursive approach, RFE systematically eliminates irrelevant or redundant features while retaining the most pertinent ones, thereby enhancing the model’s generalizability and efficacy. The essence of the RFE algorithm lies in iteratively training the model and selecting features to identify an optimal subset, thereby optimizing the model’s performance and interpretability.

The Boruta algorithm is a wrapper based on the RF algorithm (Kursa and Rudnicki, 2010). Therefore, Boruta has the advantages of the random forest algorithm, has low running time cost, and can run results without relying on parameter adjustment. It is an ensemble method where multiple independent decision trees vote for classification, classify all trees based on a given attribute and calculate the importance of all trees, i.e. the Z score, which reflects the fluctuations in accuracy between trees in the forest. When Boruta is running, it will create “shadow” attributes obtained by reshuffling the original attributes, and randomly disrupt the order of feature parameters. When calculating feature importance, the feature parameters are divided into three categories, namely Z score and significant features with a Z score higher than the “Shadow” attribute are called “Confirmed” (important), features with a Z score close to the “Shadow” attribute are called “Tentative” (potentially important), and features with a Z score significantly lower than the “Shadow” attribute are called “Rejected” (not important). In previous studies (Bai et al., 2022; Li Z et al., 2022), “Confirmed+Tentative” was used as the final result of feature selection, and no actual research was conducted to explore which feature parameter set performs better in building a model. Therefore, this study divides the Boruta algorithm screening results into two categories: “Confirmed” and “Confirmed+Tentative”, analyzes the impact of the two feature parameter sets on model construction and compares their accuracy.

In this study, the features screened by the RFE method are prefixed with R-. The feature parameter sets screened by the Boruta method are “Confirmed” and “Confirmed+Tentative”, represented by C- and CT- respectively. For example, the spectral features screened by the RFE algorithm are prefixed with R-SF means.

#### 2.5.2 Features fusion

Features fusion is a method of building a model by fusing different types of remote sensing features together (Liu et al., 2022b; Liu et al., 2023). In this study, the features fusion strategy is mainly divided into two parts: 1. First, fuse SF and TF, implement the features selection method to optimize, then build SPAD estimation models, and compare it with the model built by the fused features that did not participate in feature selection. 2. Based on the features selection strategy, the SPAD estimation model is constructed by fusing the selected feature subsets of different categories, to identify a SPAD estimation model that delivers optimal performance during the later stages of wheat growth.

### 2.6 Model development and accuracy evaluation

Based on R language version 4.1.3 (R Foundation, Vienna, Austria), the support vector machine regression (SVR) algorithm was used to predict winter wheat SPAD. The idea of SVR originated from the support vector machine. It transforms the input low-dimensional sample set into a high-dimensional space and finds a hyperplane that can be closest to all feature sample sets to implement regression, minimize structural risks, and improve sample discreteness (Smola and Schölkopf, 2004). In this study, the SPAD data set of winter wheat in each growth stage was divided into a calibration set and a validation set. The dataset was randomly sampled, 2/3 was used for model training (Calibration), and 1/3 was used to evaluate the performances (Validation) (**Table 3**). Three statistical indicators are used to test the machine learning model: the coefficient of determination (R^2^), root mean square error (RMSE), and the ratio of performance to deviation (RPD) (Bruning et al., 2019). Their calculation formulas are presented as Equations 1–3.

**Table 3:** Descriptive statistics on SPAD for the calibration and validation sets.

Stage	Dataset	Number	Min	Mean	Max	SD
Heading	Calibration	24	31.59	42.76	55.20	7.76
	Validation	12	30.17	44.82	55.31	7.23
Flowering	Calibration	24	34.38	44.62	52.74	6.42
	Validation	12	30.30	45.87	56.12	7.63
Late filling	Calibration	24	5.70	20.71	39.47	12.39
	Validation	12	6.59	22.50	44.29	12.36

Min, Mean, Max, and SD represent the minimum value, mean value, maximum value, and standard deviation of each dataset.


(1) $$R^{2}=1-\frac{\sum^{}{\left(\hat{y_{i}}-\bar{y}\right)}^{2}}{\sum^{}{\left(y_{i}-\bar{y}\right)}^{2}}\;$$

(2) $$RMSE=\sqrt{\frac{\sum_{n}^{\text{i}=1}{\left(\hat{y_{i}}-y_{i}\right)}^{2}}{\text{n}}\;}\;$$

(3) $$RPD=\frac{SD}{RMSE}\;$$

In the formula, $\hat{y_{i}}$ and $y_{i}$ are the observed values and measured values of SPAD, $\bar{y}$ is the average of SPAD observed values, n is the sample size, and *SD* is the standard deviation of reference values.

## 3 Results

### 3.1 Analysis of SPAD variation

**Figure 3:**
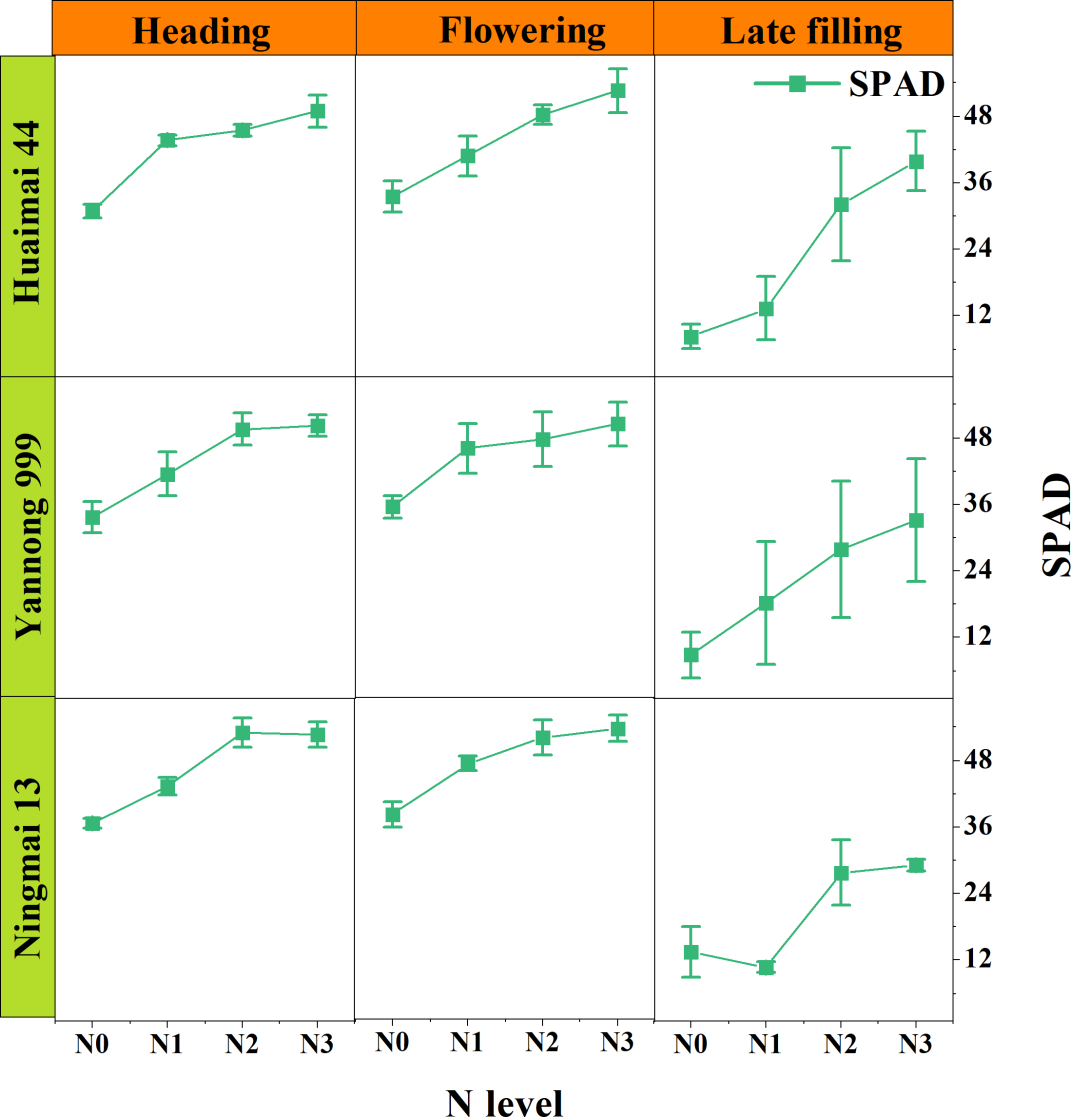
SPAD Dynamic Change Line Chart, where the nodes represent the average values of the SPAD.

**Figure 3** illustrates the dynamic changes in SPAD for wheat under different nitrogen treatments, varieties, and growth stages. As observed in the **Figure 3**, SPAD exhibits noticeable differences across various nitrogen gradient treatments, generally trending upward with increasing nitrogen application, peaking at the N3 level. The performance of SPAD varies across growth stages, initially showing a slight increase followed by a sharp decrease. Notably, the SPAD values are highest during the Flowering stage and lowest in the Late filling stage. Furthermore, no significant differences in SPAD were found among different wheat varieties, with the trends being relatively consistent across various periods and nitrogen treatments.

### 3.2 The optical features with SF and TF

**Figure 4:**
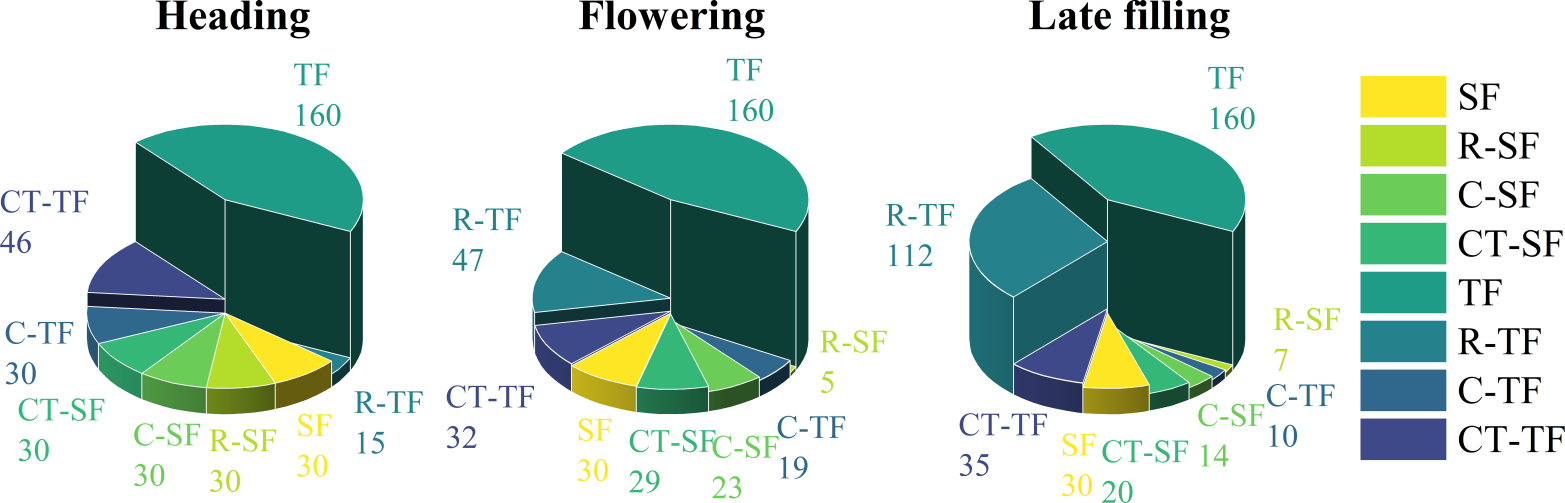
The optimal number of variables selected by Boruta and RFE features selection methods for different feature sets during different growth stages.

**Figure 5:**
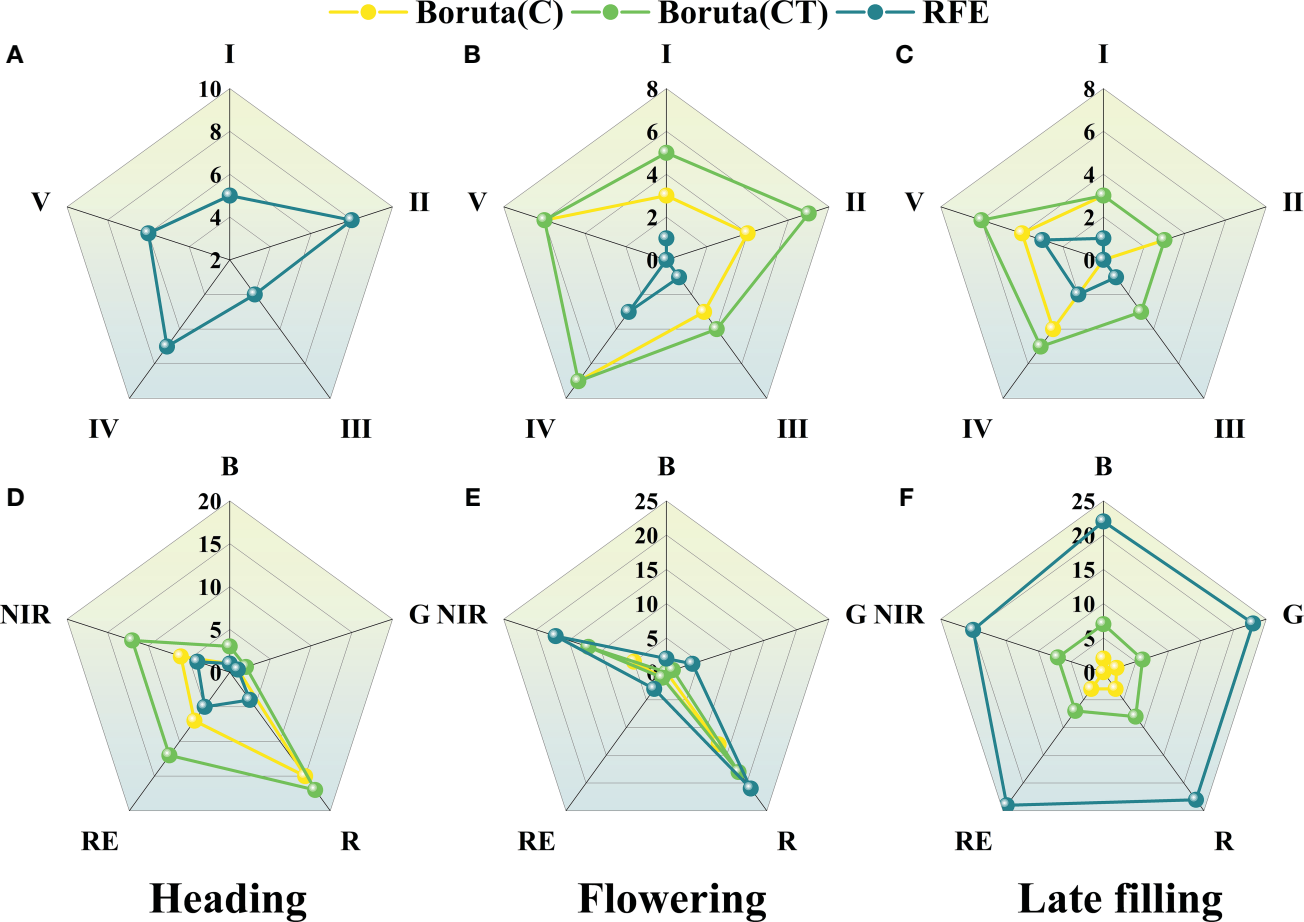
The radar chart of features selection for the three growth stages. **(A–C)** spectral features and **(D–F)** texture features.

In this study, 30 spectral features and 160 texture features were extracted from UAV multispectral images. Not all features are helpful for wheat SPAD estimation, and redundant features may affect model accuracy. Optimizing remote sensing features sensitive to SPAD using features selection strategy (**Figure 4**). As can be seen from **Figures 5A–C**, during the heading stage, three identical feature sets were selected, encompassing all five types of spectral features. In the flowering stage, spectral feature types IV and V performed the most effectively, with C-SF and CT-SF selecting all features from types IV and V, while R-SF predominantly chose type IV as the optimal variable set. In the late filling stage, types IV and V continued to excel, and the three feature sets showed a high degree of consistency in favoring spectral feature types IV and V. The commonality between spectral feature types IV and V is their composition, which includes participation from the near-infrared band.

The results of texture features band type selection for the three stages are shown in **Figures 5D–F**. During the heading stage, the R, RE, and NIR bands showed better texture performance, far exceeding the B and G bands. During the flowering stage, the trend of texture feature band types selected by the three methods was consistent, with the Red and NIR bands showing better performance. During the late filling stage, the texture feature band performances were consistent across the three selection methods, with no significant differences among the five texture feature band types. The texture performance of the R and NIR bands was relatively stable, and the number of retained features after selection was significantly higher than that of other bands, except during the late filling stage where there was no significant difference among the five bands.

### 3.3 Construction and validation of the SPAD prediction model were carried out using different strategy

#### 3.3.1 Estimation of SPAD during the late growth stage of winter wheat using features selection strategy

**Figure 6:**
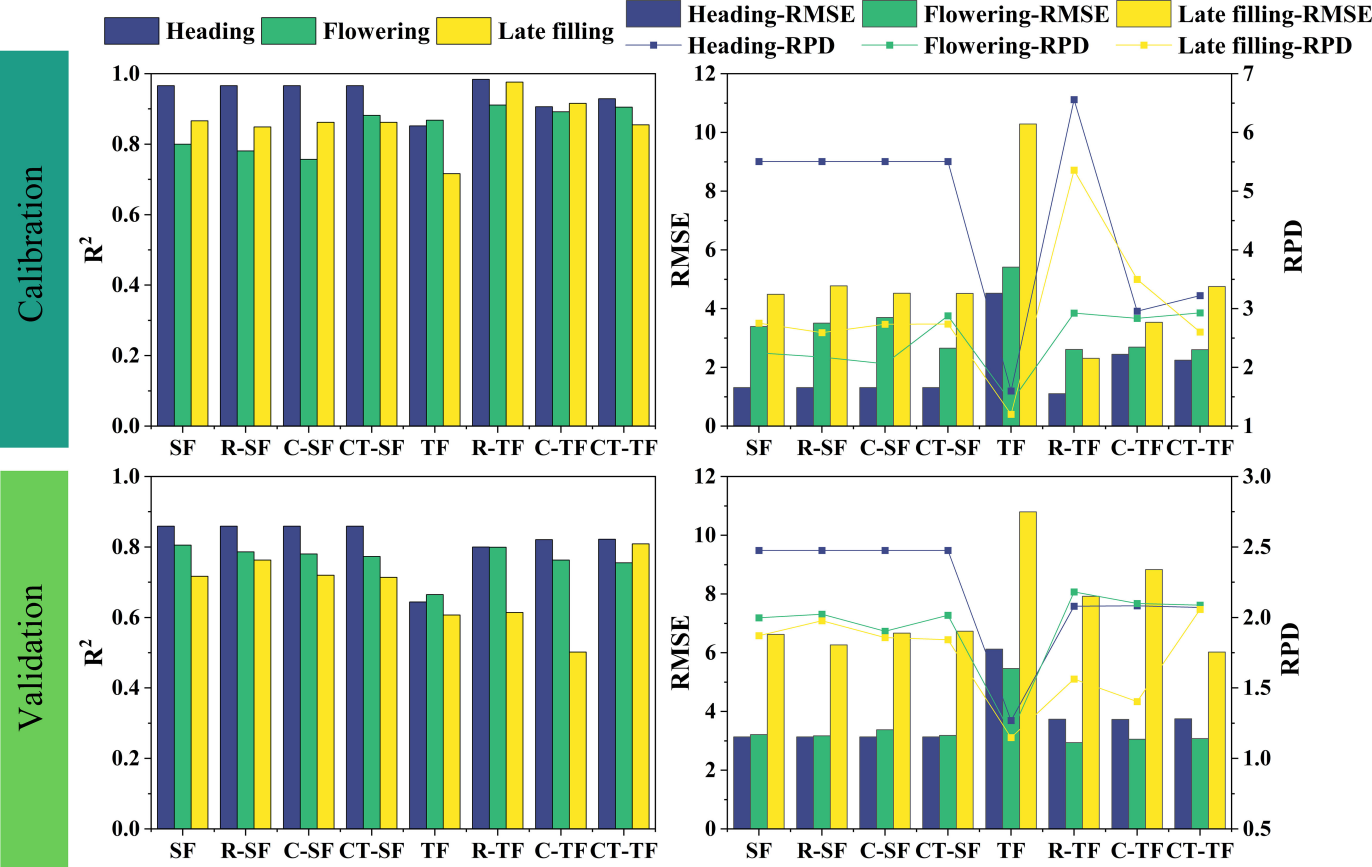
The SVR models for estimating SPAD based on different datasets in three growth stages.

In this study, SPAD estimation models were developed for the heading stage, flowering stage, and late filling stage based on spectral features and texture features extracted from UAV multispectral images. For each stage, the original feature set was included in the model construction process. A total of 24 (4*2*3) SPAD regression prediction models were built using four feature sets, two types of features (spectral features and texture features), and three growth stages (**Figure 6**).

From the perspective of model accuracy before and after feature selection, the accuracy of the models constructed using the selected dataset is generally higher compared to that of the initial dataset, but there are also some exceptions. For example, during the flowering stage, the SF dataset (Validation: R^2 ^= 0.805, RMSE=3.211, RPD=1.998) has higher accuracy than the R-SF, C-SF, and CT-SF datasets for constructing SPAD estimation models. The accuracy among the three datasets composed after features selection varies, and their performance differs in different growth stages and feature types. For example, during the heading stage, the R-TF dataset has lower accuracy (Validation: R^2 ^= 0.800, RMSE=3.732, RPD=2.080) compared to the C-TF and CT-TF datasets, while during the flowering stage, the R-TF dataset has higher accuracy (Validation: R^2 ^= 0.799, RMSE=2.941, RPD=2.181) than the C-TF and CT-TF datasets. The accuracy of the C- and CT- datasets selected by the Boruta algorithm is not consistently higher or lower.

#### 3.3.2 Estimation of SPAD during the late growth stage of winter wheat using features fusion strategy

SPAD estimation models were established for three growth stages based on spectral and texture features extracted from UAV multispectral images using a features fusion strategy, followed by feature selection. The original feature set was included in model construction at each stage, and a total of 12 (4*3) SPAD regression prediction models were constructed using four feature sets, one feature type (spectral and texture feature fusion), and three growth stages.

**Figure 7:**
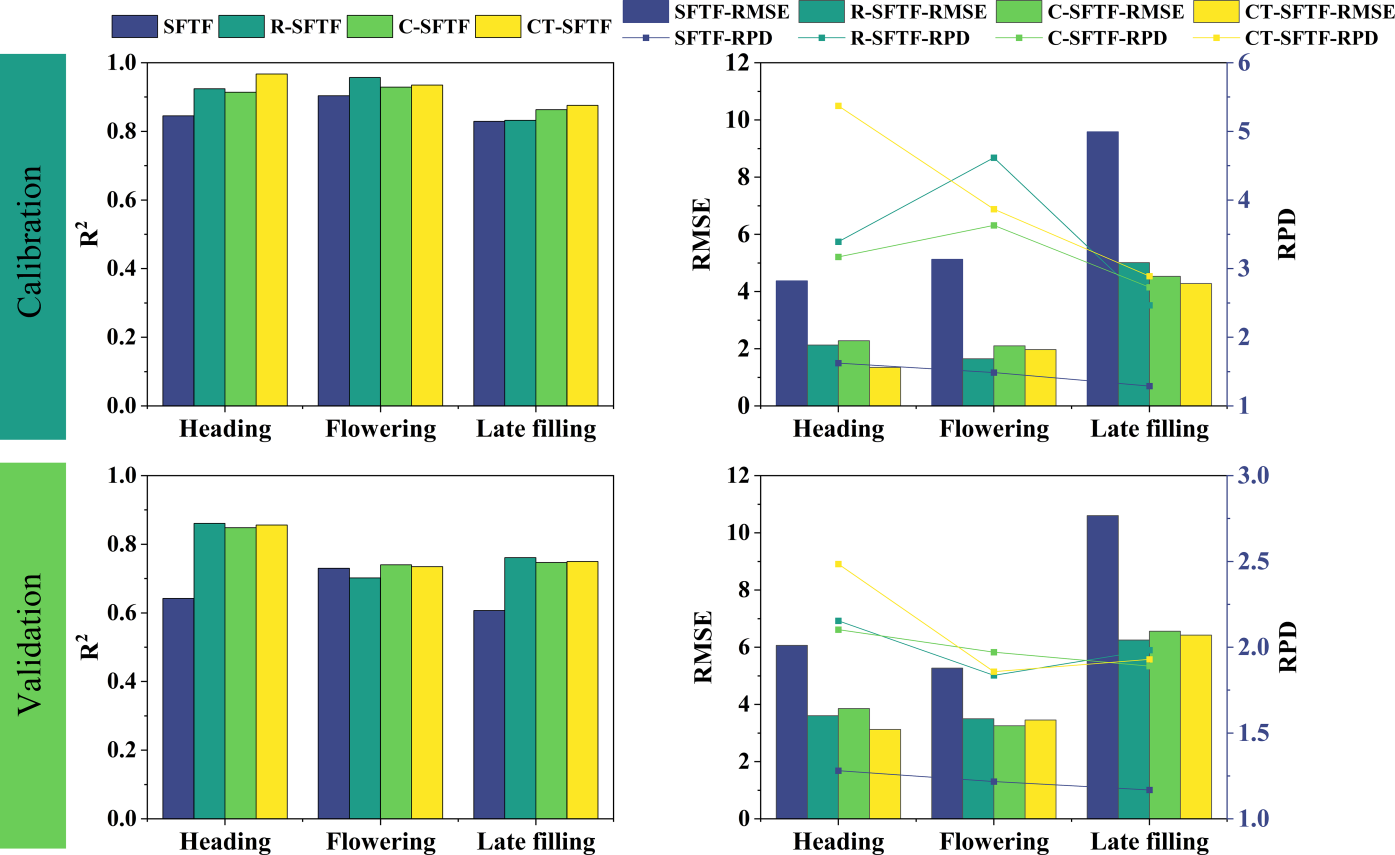
SVR models for SPAD estimation based on different fusion datasets at three growth stages.

As shown in **Figure 7**, under this feature strategy, the SPAD estimation model for winter wheat canopy at the heading stage (R-SFTF) constructed using the RFE features selection method combined with the SFTF dataset achieved the highest level of accuracy. The performance indicators of the model were as follows: Validation: R^2 ^= 0.861, RMSE=3.604, RPD=2.154. The SPAD estimation model for winter wheat at the flowering stage (C-SFTF) constructed using the Boruta features selection method combined with the SFTF dataset had the best accuracy, with specific performance indicators of Validation: R^2 ^= 0.740, RMSE=3.256, RPD=1.971. The SPAD estimation model for winter wheat at the late filling stage (R-SFTF) constructed using the RFE features selection method combined with the SFTF dataset had the best performance, with Validation: R^2^ = 0.761, RMSE=6.250, RPD=1.983.

**Figure 8:**
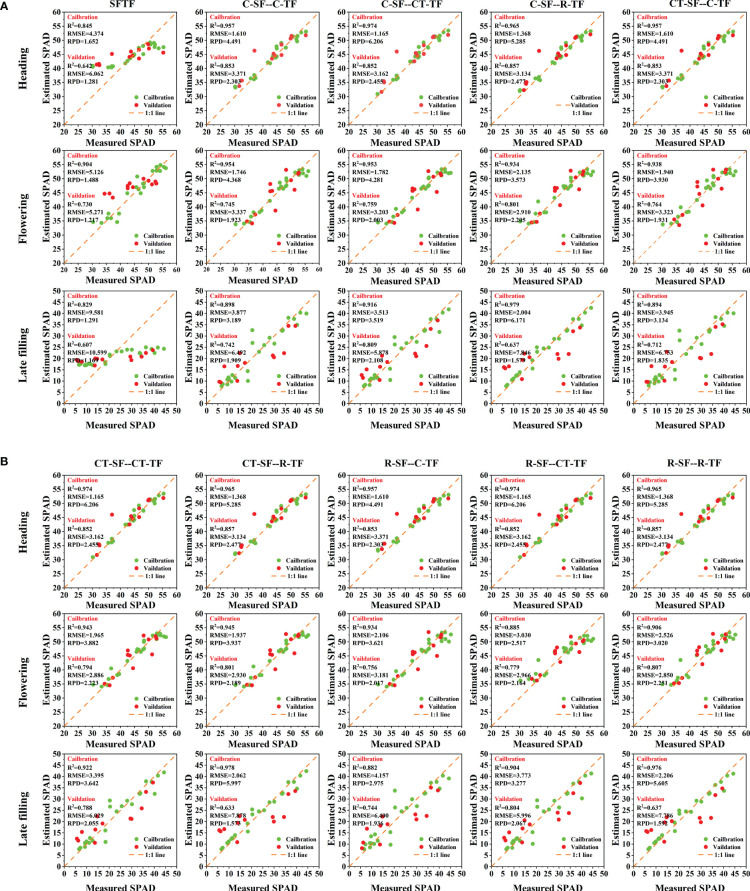
The SVR regression models constructed using the fusion dataset of selected spectral and texture features. C-, CT- and R- represent the feature set selected by the Boruta algorithm “Confirmed”, Boruta algorithm “Confirmed+Tentative” and RFE algorithm. SF represents the spectral features, TF represents the texture features. A represents the first five fusion treatments, which include SFTF, C-SF--C-TF, C-SF--CT-TF, C-SF--R-TF, and CT-SF--C-TF. B encompasses the latter five fusion treatments, consisting of CT-SF--CT-TF, CT-SF--R-TF, R-SF--C-TF, R-SF--CT-TF, and R-SF--R-TF.

On the other hand, the features fusion strategy was executed to establish SPAD estimation models for the heading stage, flowering stage, and late filling stage. For each stage, 27 SPAD prediction models (9*3) were constructed using 9 (3*3) feature sets, 1 feature type (features fusion set), and 3 growth stages. Additionally, 30 regression models were created by adding the unfiltered SFTF feature set as a comparison at each stage (**Figure 8**).

As shown in **Figure 8**, under this strategy, during the heading stage, the R-SF, C-SF, and CT-SF feature sets were the same. Therefore, there were three optimal estimation models that fused the R-TF data with these feature sets: R-SF–R-TF, C-SF–R-TF, and CT-SF–R-TF. The performance indicators of these models are as follows: Validation: R^2 ^= 0.857, RMSE=3.134, RPD=2.477. During the flowering stage, the best accuracy for canopy SPAD estimation in winter wheat was achieved by the fusion model (R-SF–R-TF) constructed using the RFE features selection method combined with the SF and TF datasets. The specific performance indicators are as follows: Validation: R^2^ = 0.807, RMSE=2.850, RPD=2.251. For the late filling stage, the best performance was observed in the fusion model (C-SF–CT-TF) constructed by combining the SF and TF datasets using the Boruta features selection method. The performance indicators for this model are as follows: Validation: R^2 ^= 0.809, RMSE=5.878, RPD=2.108.

**Figure 9:**
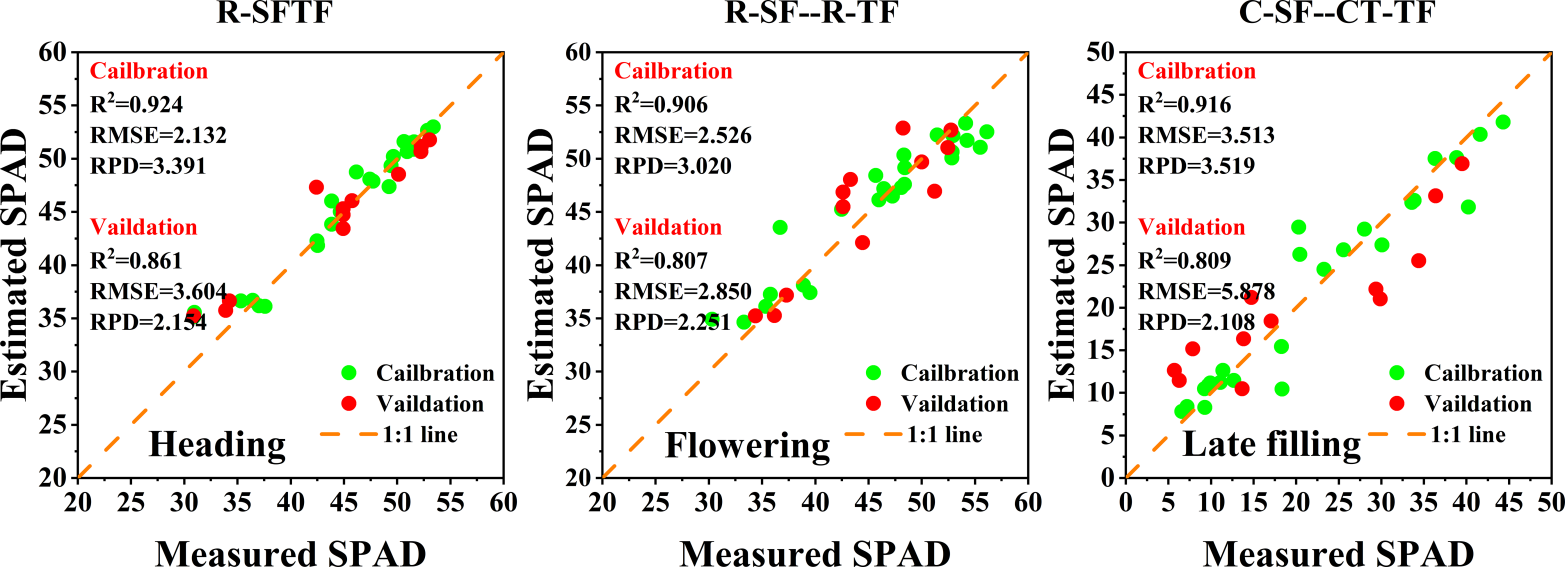
Scatter plots of the optimal SPAD estimation models.

To further analyze the accuracy of the SPAD estimation models, From the **Figure 9**, it can be observed that the optimal monitoring models for the later growth stages of winter wheat were all SPAD estimation models constructed using a dual-strategy. At each stage, the data points are clustered closely around the 1:1 line, demonstrating strong agreement between the model’s predicted values and the field-measured values, with minimal errors. These results indicate the models’ capability to accurately estimate the SPAD in winter wheat.

## 4 Discussion

### 4.1 The main spectral and texture features that affect the SPAD during the later growth stages of winter wheat

Wheat exhibits significant SPAD variation during the heading, flowering, and late filling stages, with a close relationship between SPAD and nitrogen treatment. Specifically, wheat SPAD exhibits a gradual upward trend with increased field nitrogen gradient treatments. This is because nitrogen is one of the essential nutrients for plant growth and development (Wang W et al., 2021) and a crucial component of chloroplasts in leaves. SPAD, as a measure of relative chlorophyll content in leaves, is inevitably closely related to nitrogen. During the heading and flowering stages, wheat plants absorb nitrogen from the soil through their roots. This nitrogen is then translocated to various parts of the plant, including the leaves (El-Hendawy et al., 2017). In contrast to the heading and flowering stages, SPAD in wheat drops sharply during the late filling stage. This may be due to nitrogen translocation within the plant at this time. During the filling stage, nitrogen from the leaves gradually moves into the grains, a process accompanied by a decline in photosynthetic capacity and senescence of the leaves (Bowman et al., 2015). Leaves begin to turn yellow, and the chlorophyll content decreases sharply, leading to lower SPAD values.

**Figure 10:**
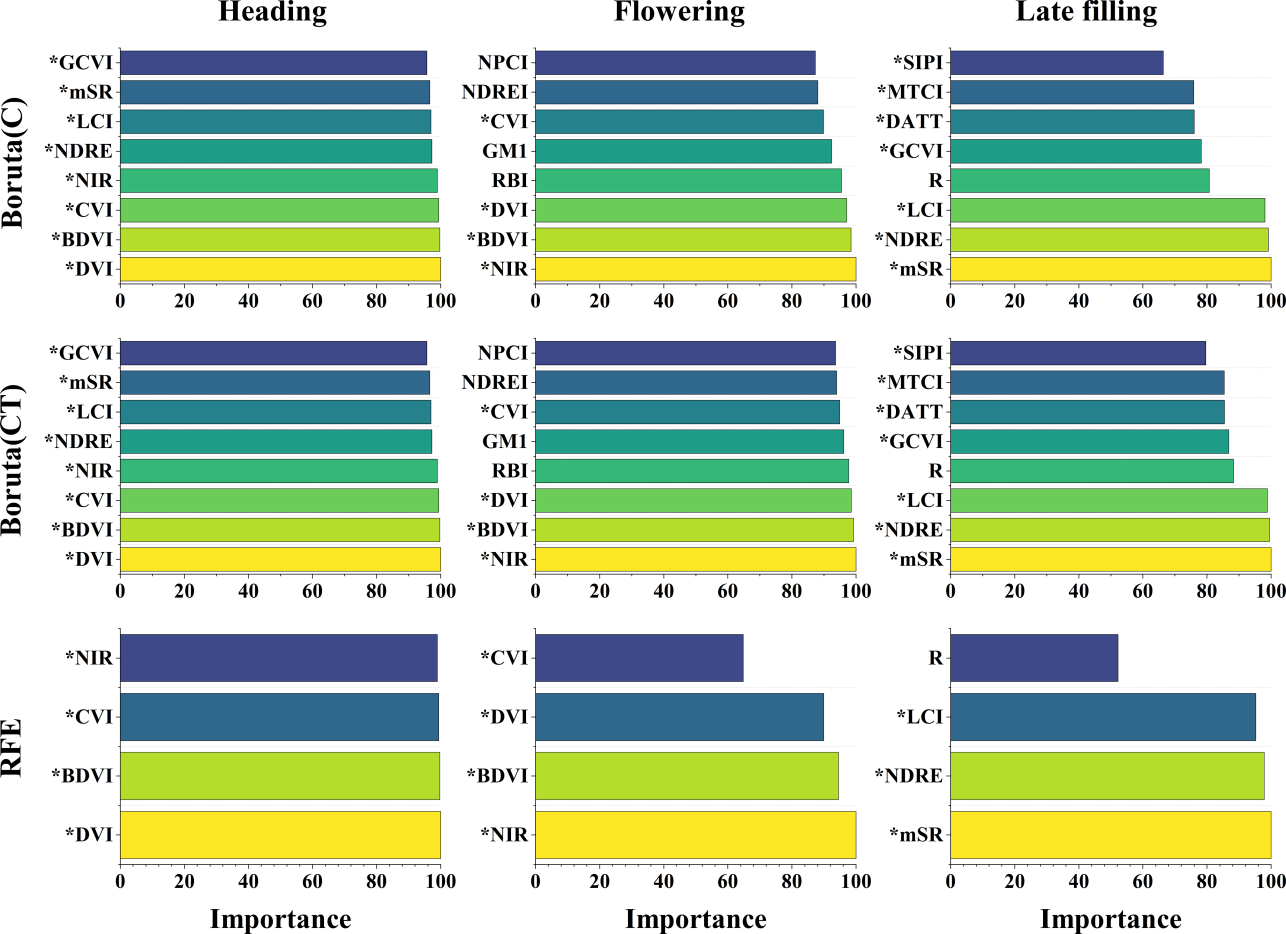
The importance of spectral features selected by different features selection methods. The Boruta algorithm displays the top 8 spectral features in terms of importance, while the RFE algorithm retains fewer features and only shows the top 4. The * symbol indicates that the spectral features involves the NIR band.

Spectral features have been verified to be closely associated with crop agronomic traits and are considered important indicators for estimating crop phenotypic information (Duan et al., 2019). It is well known that the sensitivity of spectral data to vegetation decreases during the later growth stages of crops (Zhu et al., 2023). This can be attributed to the canopy closure phenomenon and the complex spectral mixing and canopy structure effects that affect the sensitivity of spectral data to vegetation (Li R et al., 2022). Additionally, the canopy information generated by the spike emergence further exacerbates this effect (Wang et al., 2022). In this study, it was found that the NIR spectral bands combined with other spectral bands during the later growth stages of crops were the most frequently selected spectral features after optimal treatment (**Figures 5**, **10**). This finding is consistent with the conclusion reached by Yin et al. (2023), that the sensitive spectral features for estimating SPAD in winter wheat are combinations of near-infrared and other bands using three features selection methods. This may be related to the sensitivity of the NIR bands to the growth status of crops. As the growth stage progresses, nitrogen in winter wheat leaves gradually transfers to the spike organs, the leaves wilt, and the appearance of wheat plants changes, leading to a sharp change in reflectance in the near-infrared bands. Zheng et al. (2018) found that the near-infrared spectral information was most important when estimating leaf nitrogen content (LNC) using mean squared error (MSE) to evaluate variable importance. LNC is strongly correlated with SPAD, and the results of this study are similar to their conclusion, indicating the great potential of near-infrared spectral data for estimating SPAD during the later growth stages of winter wheat.

**Figure 11:**
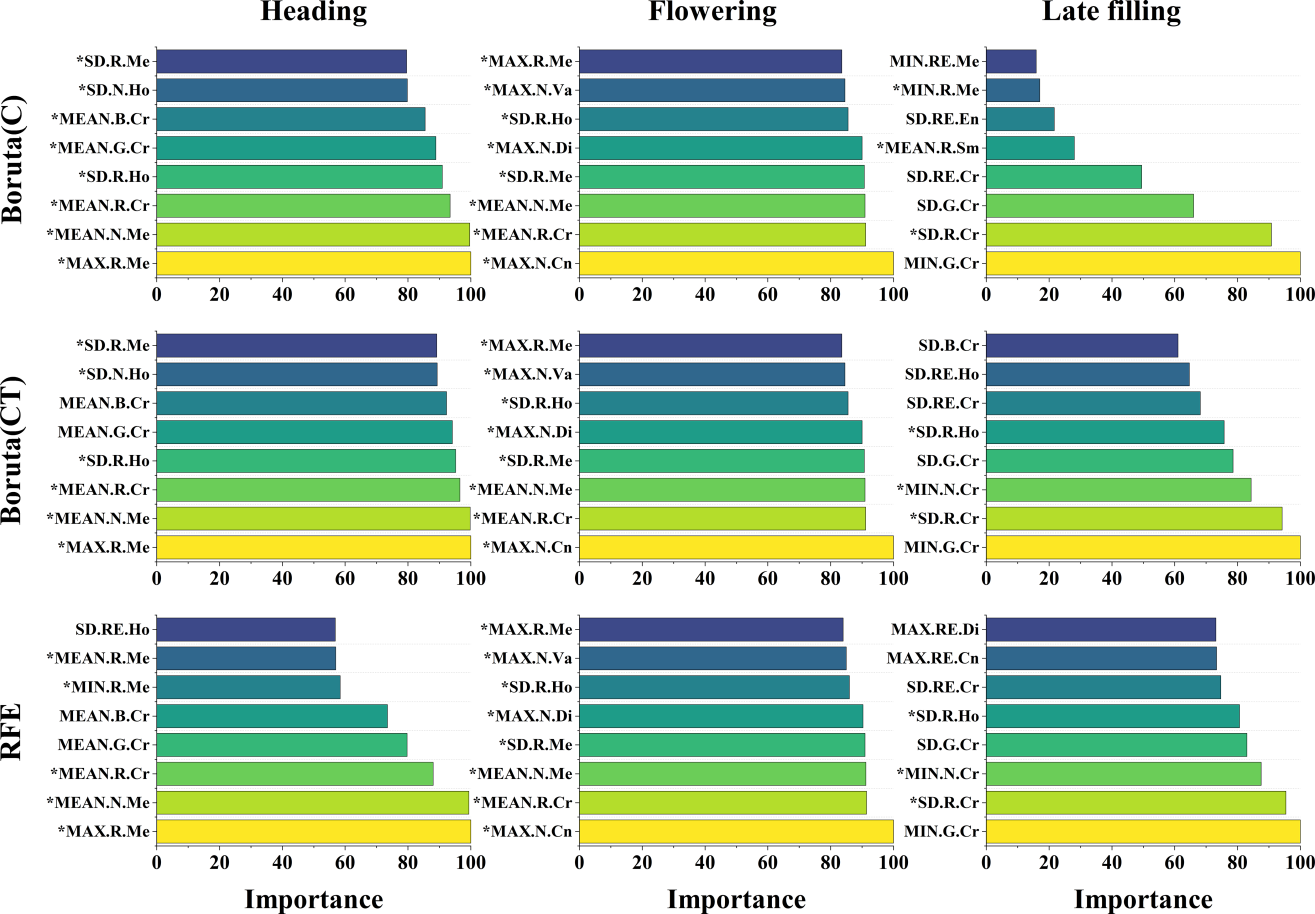
The importance of texture features selected by different features selection methods. The Boruta and RFE algorithms display the top 8 texture features in terms of importance. The * symbol indicates that the texture features belongs to the red and NIR bands.

**Figure 12:**
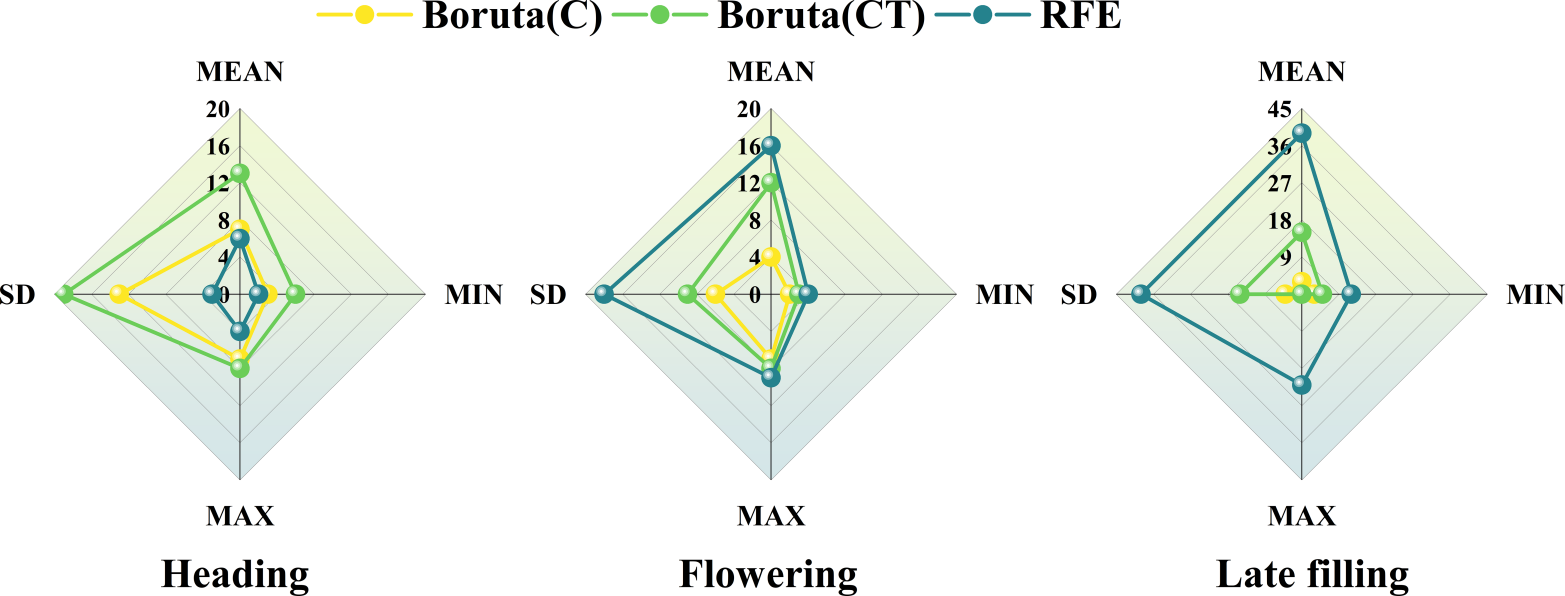
The statistical indices of the retained texture features at the three growth stages of wheat.

In this study, the texture features of the red and near-infrared bands maintained a high sensitivity to SPAD information during the reproductive growth stage of wheat (**Figures 5**, **11**). This is consistent with the conclusion reached by Bai et al. (2022), that the texture features of the red band can better predict soybean yield compared to the blue and green bands, and with the conclusion reached by Lu et al. (2021), that extracted texture features from the near-infrared band obtained by multispectral sensors are more effective in predicting potassium content in rice plants than other bands. This may be because the texture features of the red and near-infrared bands can better describe the spatial distribution changes of the crop canopy, providing valuable information for estimating crop phenotypes. It also demonstrates the feasibility of using UAV equipped with RGB sensors (including the red band) or multispectral sensors (including the red and near-infrared bands) to monitor crop phenotypic information using texture features. Furthermore, previous studies that used texture features to construct crop phenotyping models mostly considered the relationship between the mean (MEAN) of texture feature indices and crop phenotypes (Fu et al., 2021; Liu et al., 2022a; Luo et al., 2022; Zhou et al., 2022), while rarely exploring other statistical indices. In this study, further analysis of statistical indices of texture features (**Figures 11**, **12**) extracted four different statistical indices based on GLCM features for each sample area: MEAN, SD, MAX, and MIN. The commonly used MEAN feature consistently maintained a leading position among the four statistical indices (**Figure 12**), but its importance gradually decreased as the growth stage progressed (**Figure 11**). The changing trend of importance for other indices was opposite to MEAN. From the heading stage to the late filling stage, their importance gradually increased and reached a peak in the late filling stage, with SD showing the best trend. This indicates that other statistical indices also have the potential to estimate SPAD in wheat, especially SD. This is consistent with the conclusion reached by Bai et al. (2022), that using texture features incorporating SD information achieved good accuracy and precision in predicting early soybean yield. The results of this study indicate that the SD information of texture features also has the potential to estimate crop SPAD.

### 4.2 The impact of features selection strategy on SPAD estimation

**Figure 13:**
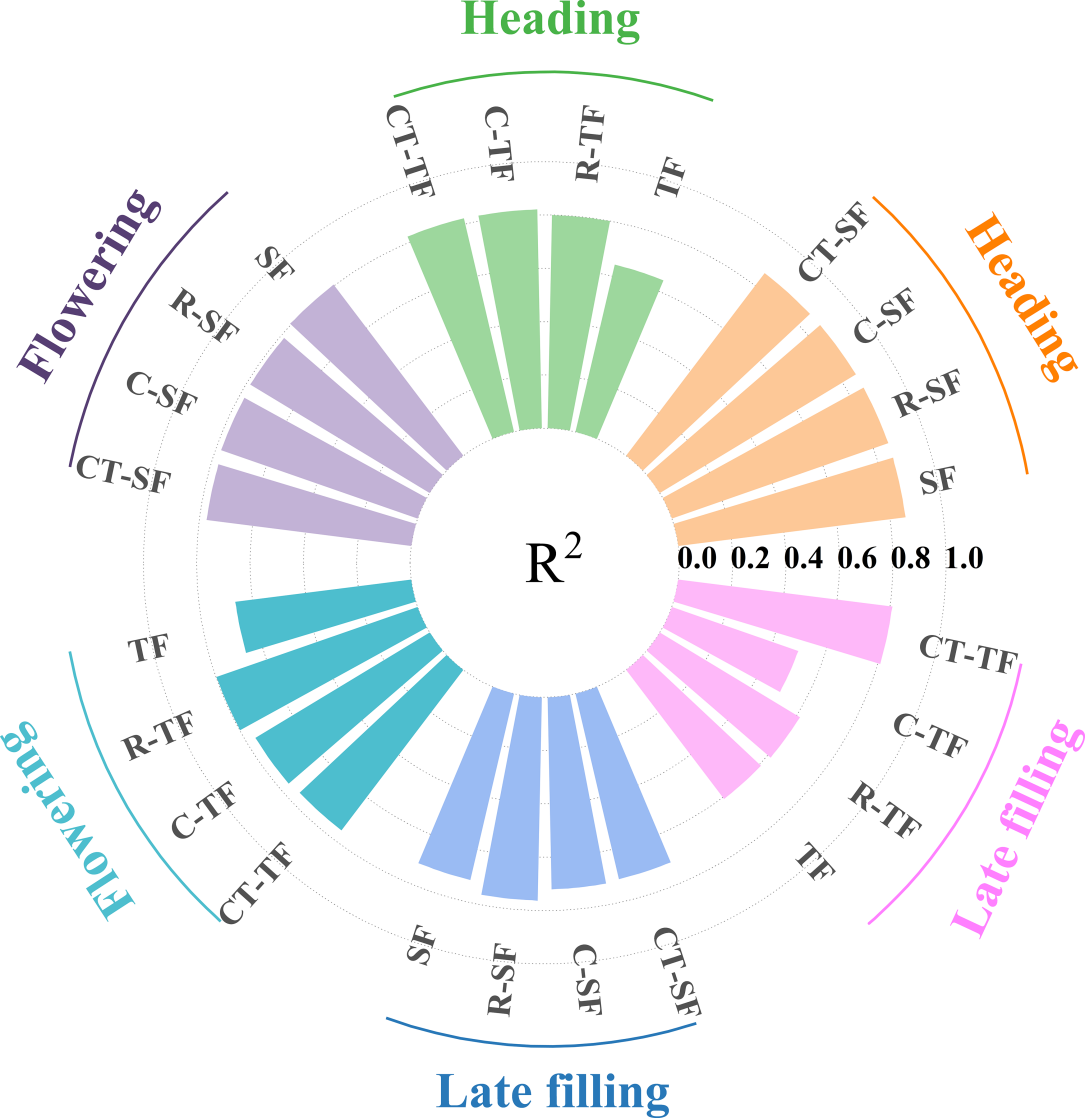
The comparison of R^2^ values for the validation accuracy of winter wheat SPAD estimation models constructed using features selection methods to select suitable remote sensing features at three growth stages.

Features selection methods play a significant role in machine learning and can directly impact the predictive model (Kursa and Rudnicki, 2010). When estimating crop phenotypes using remote sensing features, selecting appropriate remote sensing features through features selection strategy can improve model performance and eliminate interference (Lin et al., 2021; Xu et al., 2023). In this study, two features selection methods, Boruta and RFE, were used to select optimal spectral and texture features and build models. Additionally, a comparison of model accuracy before and after features selection was conducted to further analyze the mechanism of features selection methods and select suitable methods for crop phenotype studies. From **Figure 13**, it can be observed that the accuracy of the SPAD estimation model constructed using the subset of variables after features selection is generally better than that of the original variable set. However, there are also some exceptions, as the Boruta method shows a slight decrease in model accuracy (**Figure 13**, Late filling, TF and C-TF). This may be related to the principle of the Boruta method. Boruta cannot identify and explore features with low individual value but high combined value, leading to the removal of low-value features and reducing or eliminating the high value generated by feature combinations, thus resulting in decreased model accuracy. Furthermore, Boruta focuses on selecting all features related to SPAD (Kursa and Rudnicki, 2010), overlooking the relationship between features, which may lead to multicollinearity among features and subsequently reduce model performance.

Therefore, features selection techniques should not only consider the relationship between features and SPAD but also take into account the relationship between features. RFE does this well by continuously combining subsets of variables to build models in order to find the optimal variable set with the minimum RMSE. For example, Wang J et al. (2021) used two features selection methods, RF and r, to construct RF and SVR models for estimating winter wheat SPAD, and compared them with the RFE features selection method. They found that the RFE features selection method combined with RF and SVR models had higher predictive accuracy. Lin et al. (2021) compared three features selection methods, Boruta, Sequential Forward Selection (SFS), and Permutation Importance-Recursive Feature Elimination (PI-RFE), and found that the PI-RFE method had the best dimensionality reduction effect on multidimensional feature sets, greatly improving the accuracy of FVC inversion. In this study, whether it was at the heading stage or flowering stage, the RFE features selection method combined with the SVR algorithm provided the best SPAD prediction model accuracy. In the late filling stage, the RFE-SVR combination still showed high SPAD estimation ability (**Figures 7**, **8**). Overall, compared with the Boruta method, the RFE combined with the SVR algorithm can achieve higher model accuracy and stronger model performance. RFE takes into account the interrelationships between features and between features and SPAD, thereby improving the performance of prediction model.

### 4.3 The impact of features fusion strategy on SPAD estimation

Features fusion strategy involve merging different types of features into a more optimal feature set, combining features with different features to construct models, and have the potential to mitigate the impact of spectral saturation effects, enhance model performance and increase the interpretability of prediction models (Zhou et al., 2022; Sun et al., 2023). This explains why the optimal SPAD estimation models in the late growth stage of winter wheat are constructed using features fusion strategy. This study comprehensively reports the essence of the impact of features fusion strategy on model performance through two features fusion approaches. When only implementing features fusion strategy (**Table 4**), it was found that not all feature fusions lead to improved accuracy. Blindly combining features together to build models may have a counterproductive effect, causing data redundancy and reducing model performance (Son et al., 2015; Li et al., 2020). This conclusion is in line with the discoveries of Zhou L et al. (2023), who observed a decrease in model accuracy when agronomic practice information (API) was fused with spectral and texture features to construct a rice yield estimation model. The essence of the impact of features fusion strategy on model accuracy lies in the contribution of multi-characteristic features to the model and the interactions between features. This also explains why there are differences in predictive model results when different variables are fused (**Figure 8**). Similar to the findings of Liu J et al. (2022), who estimated rice nitrogen use efficiency (NUE) using different numbers of spectral features and concluded that increasing the number of variables does not necessarily improve model accuracy, this study also discovered that there is no essential correlation between model accuracy and the quantity of features (**Table 4**; **Figures 4**, **6**–**8**, **12**, **13**). Adding variables does not necessarily make the model more robust, and features selection strategy are powerful means to address these issues.

**Table 4:** Constructing SPAD estimation model using the dataset of SF,TF and SFTF.

Stage	Dataset (original)	Number	Validation		
			R^2^	RMSE	RPD
Heading	SF	30	0.859	3.135	2.476
	TF	160	0.644	6.119	1.269
	SFTF	190	0.642	6.062	1.281
Flowering	SF	30	0.805	3.211	1.998
	TF	160	0.665	5.463	1.175
	SFTF	190	0.730	5.271	1.217
Late filling	SF	30	0.717	6.624	1.871
	TF	160	0.607	10.794	1.148
	SFTF	190	0.607	10.599	1.169

SF represents initial spectral features, TF represents initial texture features, and SFTF represents the fused feature set obtained by combining the initial spectral features and initial texture features.

After combining features fusion strategy with features selection strategy, the stability, accuracy, and robustness of the SPAD estimation models for winter wheat are at the forefront (**Figure 9**). As the growth stage progresses, the improvement in model accuracy gradually increases. The highest increase in model accuracy is observed in the late filling stage, where compared to models using only initial spectral or texture features, R^2^_Val_ increased by 0.092 to 0.202, RMSE_Val_ decreased by 0.076 to 4.916, and RPD_Val_ increased by 0.237 to 0.960. Sun et al. (2023) developed maize LAI estimation model using SVM, RF, BPNN, and PLSR algorithms by selecting five spectral features and three texture indices through correlation analysis. The results showed that the SVM algorithm with multi-variable fusion achieved the highest accuracy. Compared to SVM models using only spectral or texture features as input variables, R^2^_Val_ increased by 0.023 to 0.192, RMSE_Val_ decreased by 0.015 to 0.036, and RPD_Val_ increased by 0.074 to 0.746. Ma et al. (2022) used four features selection methods, including correlation coefficient, MIC, RF, and RFE, to select three spectral features and three texture features to construct a cotton yield estimation model. The results showed that the RFE_ELM model based on the fusion of spectral and texture features achieved the highest accuracy, with an increase in R^2^_Val_ of 0.073 to 0.187 compared to the non-fusion model. The findings of this study surpass those of prior research, indicating that the combination of features fusion strategy and features selection strategy is an effective method to mitigate the impact of spectral saturation effects and improve the accuracy of crop phenotype estimation, fully tapping into the potential of multi-characteristic features in estimating crop phenotypes.

### 4.4 Limitations and future research perspectives

This study conducted field experiments on winter wheat using three locally stable and high-yielding varieties (Huaimai 44, Yannong 999, and Ningmai 13) and four nitrogen fertilizer application levels (N0-N3: 0, 100, 200, 300 kg/ha). In the future, we will systematically supplement the dataset and conduct field experiments on more varieties and nitrogen fertilizer treatments. At the same time, because of the swift advancement in sensor technology, there are still limitations in building SPAD prediction models relying solely on single sensor data. In the future, we plan to incorporate RGB and hyperspectral data into the SPAD prediction model. The machine learning algorithms employed in this study are relatively single, and in the future, we will introduce multiple algorithms for comparison and select the most suitable algorithm for SPAD estimation. Furthermore, the use of multi-source remote sensing information and background effect removal can also improve the accuracy of crop phenotype estimation based on UAV. In the future, we will combine them to further improve the accuracy of SPAD estimation for winter wheat. Although the combination of features fusion strategy and features selection strategy performs well in SPAD estimation during the later growth stage of wheat, the results of this study only come from small-scale experiments. In the future, we will conduct large-scale integrated winter wheat SPAD remote sensing monitoring research using satellite and UAV platforms.

## 5 Conclusion

This study demonstrates that spectral and texture features have good potential for monitoring the SPAD status of winter wheat in the reproductive stage. Spectral features in the near-infrared band can fully capture the spectral differences of wheat SPAD in the reproductive growth stage, while texture features in the red and near-infrared bands are more sensitive to wheat SPAD. Among the SPAD estimation models under different strategy, the SVR model combining features selection and features fusion strategy had the highest accuracy, and compared with the SVR model that only used initial spectral or texture features as input, the stability of the model was improved. As the growth stage progresses, the enhancement of model accuracy by this method becomes more significant, with the greatest improvement observed during the late filling stage. R^2^_Val_ increased from 0.092 to 0.202, RMSE_Val_ decreased from 0.076 to 4.916, and RPD_Val_ increased from 0.237 to 0.960. This study shows that UAV remote sensing technology combined with features selection and fusion strategy has good prospects for monitoring winter wheat SPAD in the reproductive growth stage, and can accomplish precise monitoring of winter wheat growth under different varieties and nitrogen treatments, providing scientific guidance and theoretical support for fine management of field crops nutrition.

## Data availability statement

The original contributions presented in the study are included in the article/supplementary material. Further inquiries can be directed to the corresponding authors.

## Author contributions

XS: Conceptualization, Data curation, Formal analysis, Methodology, Resources, Writing – original draft, Writing – review & editing, Software. YN: Data curation, Formal analysis, Writing – review & editing. HS: Writing – review & editing. AH: Data curation, Methodology, Software, Writing – review & editing. HY: Data curation, Formal analysis, Writing – review & editing. YZ: Conceptualization, Data curation, Methodology, Writing – review & editing. JLi: Data curation, Methodology, Software, Writing – review & editing. WW: Data curation, Methodology, Writing – review & editing. HW: Supervision, Writing – review & editing. QM: Writing – review & editing, Formal analysis, Methodology. JLiu: Methodology, Writing – review & editing, Funding acquisition, Software, Writing – original draft. XL: Methodology, Software, Writing – original draft, Writing – review & editing, Funding acquisition, Supervision, Validation. YA: Supervision, Validation, Writing – review & editing.
